# Accelerated Hypofractionated Magnetic Resonance Guided Adaptive Radiation Therapy for Ultracentral Lung Tumors

**DOI:** 10.3390/tomography10010013

**Published:** 2024-01-17

**Authors:** Alonso La Rosa, Kathryn E. Mittauer, Nema Bassiri, Amy E. Rzepczynski, Michael D. Chuong, Sreenija Yarlagadda, Tugce Kutuk, Nicole C. McAllister, Matthew D. Hall, Alonso N. Gutierrez, Ranjini Tolakanahalli, Minesh P. Mehta, Rupesh Kotecha

**Affiliations:** 1 Department of Radiation Oncology, Miami Cancer Institute, Baptist Health South Florida, Miami, FL 33176, USA; kathrynm@baptisthealth.net (K.E.M.); nema.bassirigharb@baptisthealth.net (N.B.); amyrz@baptisthealth.net (A.E.R.); michaelchu@baptisthealth.net (M.D.C.); sreenija.yarlagadda@baptisthealth.net (S.Y.); tugcek@baptisthealth.net (T.K.); nicolemca@baptisthealth.net (N.C.M.); matthewha@baptisthealth.net (M.D.H.); alonsog@baptisthealth.net (A.N.G.); ranjinit@baptisthealth.net (R.T.); mineshm@baptisthealth.net (M.P.M.); 2 Department of Radiation Oncology, Herbert Wertheim College of Medicine, Florida International University, Miami, FL 33199, USA; 3 Department of Translational Medicine, Herbert Wertheim College of Medicine, Florida International University, Miami, FL 33199, USA

**Keywords:** lung tumor, ultracentral, MRgART, radiation therapy, adaptive

## Abstract

Radiotherapy for ultracentral lung tumors represents a treatment challenge, considering the high rates of high-grade treatment-related toxicities with stereotactic body radiation therapy (SBRT) or hypofractionated schedules. Accelerated hypofractionated magnetic resonance-guided adaptive radiation therapy (MRgART) emerged as a potential game-changer for tumors in these challenging locations, in close proximity to central organs at risk, such as the trachea, proximal bronchial tree, and esophagus. In this series, 13 consecutive patients, predominantly male (*n* = 9), with a median age of 71 (range (R): 46–85), underwent 195 MRgART fractions (all 60 Gy in 15 fractions) to metastatic (*n* = 12) or primary ultra-central lung tumors (*n* = 1). The median gross tumor volumes (GTVs) and planning target volumes (PTVs) were 20.72 cc (R: 0.54–121.65 cc) and 61.53 cc (R: 3.87–211.81 cc), respectively. The median beam-on time per fraction was 14 min. Adapted treatment plans were generated for all fractions, and indications included GTV/PTV undercoverage, OARs exceeding tolerance doses, or both indications in 46%, 18%, and 36% of fractions, respectively. Eight patients received concurrent systemic therapies, including immunotherapy (four), chemotherapy (two), and targeted therapy (two). The crude in-field loco-regional control rate was 92.3%. No CTCAE grade 3+ toxicities were observed. Our results offer promising insights, suggesting that MRgART has the potential to mitigate toxicities, enhance treatment precision, and improve overall patient care in the context of ultracentral lung tumors.

## 1. Introduction

The increasing role of radiation therapy (RT) in the management of patients diagnosed with oligometastatic non-small cell lung cancer (NSCLC) presents a promising alternative to systemic therapy alone [1,2,3]. This shift is particularly relevant for patients with oligometastatic disease [4]. Stereotactic body radiation therapy (SBRT) is a primary approach for irradiating metastatic lesions, though it does come with certain limitations. For example, it is primarily applicable to limited treatment volumes (≤5 cm) away from critical organs at risk (OARs). Consequently, when addressing the primary lung site and associated regional lymphadenopathy, conventionally fractionated schedules are still frequently employed [5].

Treatment-related toxicities have been compared, with similar results in previous retrospective studies, between moderately hypofractionated schedules (>3 Gy per fraction, total dose ~45 Gy) and conventionally fractionated ones (1.8 to 2 Gy per fraction, total dose 54–60 Gy) [6]. Conversely, more aggressively hypofractionated schedules are linked to higher rates of treatment-related adverse events. Notably, a recent phase 3 randomized trial evaluated a high-dose hypofractionated schedule (60 Gy in 15 fractions) with modern image-guided radiation therapy (IGRT) against a conventionally fractionated IGRT schedule (60 Gy in 30 fractions) for stage II-III NSCLC and revealed an increased incidence of grade 2+ toxicities with hypofractionation [7]. These toxicities were primarily due to damage to central OARs, such as the proximal bronchial tree (PBT) and esophagus, resulting in increased dyspnea (Common Terminology Criteria for Adverse Events (CTCAE) v5.0 grade 2+ 10% vs. 4.3%) and esophagitis (CTCAE v5.0 grade 2+ 24% vs. 10.9%). These data underscore the significant challenges in utilizing aggressively hypofractionated schedules in large central thoracic lesions, and this is particularly challenging when associated mediastinal adenopathy needs to be addressed.

In this context, magnetic resonance-guided adaptive radiation therapy (MRgART) emerges as an innovative solution, offering a comprehensive approach to facilitate hypofractionation, which is especially important for patients with metastatic disease. MRgART provides several benefits over other CT-based RT platforms. Firstly, magnetic resonance (MR) technology provides superior soft tissue visualization, including detailed imaging of mediastinal anatomy [8]. Secondly, MRgART offers continuous, real-time cine-MR imaging, facilitating gated RT and reducing the potential for high-dose overlap with critical central OARs like the PBT or trachea. Thirdly, MRgART enables daily dosimetric evaluation by comparing the original treatment plan with the anatomy on the treatment day (predicted plan), thus assessing dosimetric variations due to interfractional differences, such as the esophagus position. Furthermore, it allows for the creation of an on-table plan (adaptive on-line plan) tailored to the specific new anatomy, if necessary. Notably, our institution has previously outlined our workflow for accelerated hypofractionated MRgART for ultracentral lung tumors [5]. This report represents the inaugural investigation into a consecutive cohort of patients with ultra-central lung tumors treated with MRgART to report treatment characteristics, dosimetry evaluations at initial plan development and daily treatment imaging, and early outcomes.

## 2. Materials and Methods

### 2.1. Patient Selection

We included all patients with ultracentral lung tumors, institutionally defined as those where the planning target volume (PTV) overlaps the trachea, mainstem bronchi, or esophagus [9], who underwent accelerated hypofractionated MRgART to a total dose of 60 Gy in 15 fractions from June 2020 to May 2023. The rationale for this ultracentral definition stems from its proximity to critical structures and the potential impact on treatment planning and outcomes. It is important to acknowledge the potential variations and limitations in definitions, which will be further discussed. This retrospective analysis was approved by the institutional ethics committee.

### 2.2. Accelerated Hypofractionated MRgART

#### 2.2.1. Radiation Therapy Simulation

Patients were simulated in the supine position, typically with at least the ipsilateral arm up. A simulation of true fast imaging with steady-state free precession (TrueFISP) MR sequence was acquired on a 0.35 T MRIdian (ViewRay Inc., Denver, CO, USA) linear accelerator in mid-inspiration breath hold. On the same day, a CT simulation scan was acquired in similar breath-holding conditions for dosimetric calculation purposes [10]. No contrast was utilized in any case.

#### 2.2.2. Targets and Organ-at-Risk Contouring

The MRIdian TrueFISP sequence (1.5 mm in-plane spatial resolution with 3 mm slice thickness) was performed for the simulation and used for the target volume and OAR delineation. The gross tumor volume (GTV) was defined as the tumor and lymph nodes visualized on the TrueFISP sequence. A 5 mm margin expansion was added to the GTV to create the PTV. Since the simulation and treatments were performed in breath hold, no internal target volume (ITV) expansion was required in any patients. Normal OARs include the spinal cord, heart, lungs, great vessels, esophagus, PBT, and trachea.

#### 2.2.3. Treatment Planning and Delivery

The total prescribed dose was 60 Gy in 15 fractions, with the clinical target goal of 99% and 95% of the GTV and PTV, respectively, to be covered by 100% of the prescription dose. Our institutional treatment plan directives (target coverage and OAR constraints) have been described previously [5]. Baseline intensity-modulated radiation therapy (IMRT) step-and-shoot treatment plans with a median of 18 beams (range: 15–21) and 88 segments (range: 56–126) were generated based on the simulation breath-hold MR scan. As mentioned, for electron density calculation, a CT scan was performed in the same session for dose calculation purposes.

Before each treatment fraction (once daily; five days a week), a new mid-inspiration breath-hold MR TrueFISP sequence was repeated (in the same simulation scan conditions). Target volumes were rigidly registered and recontoured by the treating physician. OARs underwent deformable registration, and those within 3 cm radially and 2 cm cranio-caudal from the PTV were recontoured by the treating physician. A predicted dose was calculated for the targets and OARs with the new anatomic changes. Re-optimization was pursued if there was target undercoverage, if the dose to critical surrounding OARs exceeded preset tolerances, or both. All patients underwent a secondary Monte Carlo calculation for independent fluence quality assurance.

### 2.3. Analysis of Dosimetric and Clinical Outcomes

All dosimetric values for targets and OARs were collected from the original plans, both for the daily predicted and adapted plans. Patients were followed for clinical outcomes and treatment-related toxicities. Reported toxicities were scored using CTCAE v5.0.

## 3. Results

### 3.1. Patient Characteristics

**Figure 1:**
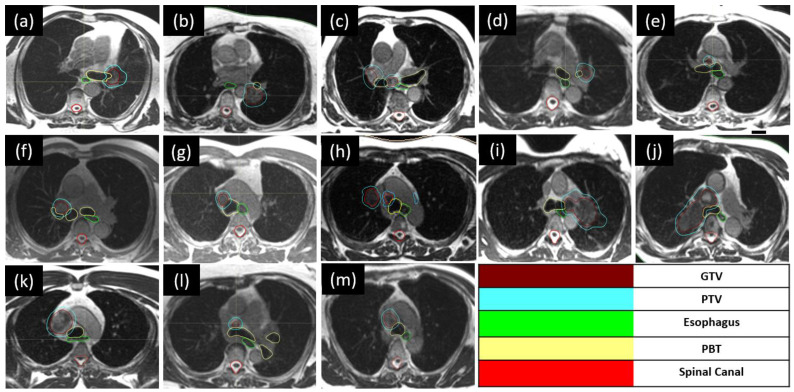
(**a**–**m**). Axial slices for the consecutive cases treated with accelerated hypofractionated MRgART (60 Gy in 15 fractions). All displayed MR TrueFISP sequences demonstrate the proximity of the target volumes (blue) with the central thoracic organs at risk (green, yellow). GTV = gross tumor volume. PTV = planning target volume. PBT = proximal bronchial tree.

Thirteen consecutive patients were treated with an accelerated hypofractionated MRgART (60 Gy in 15 fractions) over 195 fractions (Figure 1). Patients and treatment characteristics are summarized in Table 1. The median age of patients was 71 years (range, 46–85), and the majority were male (9 versus 4 females). Treatment indications included lung/mediastinum oligometastases (11 for oligoprogressive disease and 1 for oligorecurrent disease) and early-stage NSCLC (1 patient; 1 tumor) based on either pathological or clinical diagnosis. For metastatic patients, the primary histology was adenocarcinoma (*n* = 8), followed by small cell carcinoma (*n* = 2), squamous cell carcinoma (*n* = 1), carcinosarcoma (*n* = 1), and melanoma (*n* = 1). Eight patients received concurrent systemic therapies, including immune therapy (four), chemotherapy (two), and targeted therapy (two). All patients had a good performance status prior to treatment, with an Eastern Cooperative Oncology Group (ECOG) score of 1.

### 3.2. MR Linac Treatment

For all thirteen patients, the median breath-hold GTVs and PTVs were 20.72 cc (range, 0.54–121.65 cc) and 61.53 cc (range: 3.87–211.81 cc), respectively. The median PTV coverage by the prescription dose (PD) was 95.0% in baseline plans. The maximum dose, as a percentage of the PD, was a median of 111.4% (66.9 Gy; range, 65.4–83.8 Gy) within the GTV and PTV.

The median duration of a MRgART session, as measured from the patient entering the treatment room to the end of treatment delivery, was 48 min (5th to 95th percentile, 30–74 min). The median gated beam-on time was 14 min (5th to 95th percentile, 9–27 min). Treatment was completed as scheduled for all patients, without any delays.

### 3.3. Online Plan Adaptation

**Figure 2:**
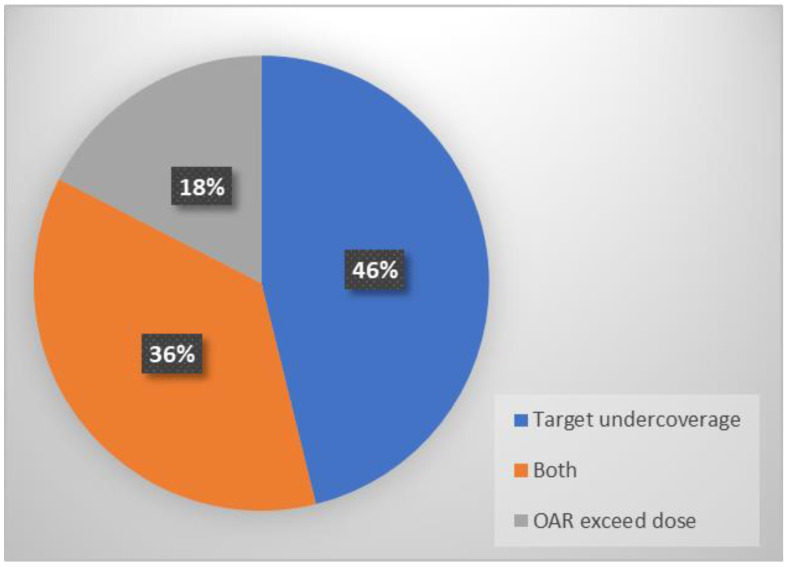
Pie chart showing the rationales for online adaptation for all 195 treatment fractions.

All 195 fractions were delivered using on table-reoptimized treatment plans. The rationale for proceeding with online adaptation was recorded for each fraction based on the evaluation of the target coverage and/or OAR predicted doses exceeding the constraints from the original plan in the new anatomical distribution (structure deformations). As shown in Figure 2, the primary rationale for online adaptation was GTV/PTV undercoverage (46% of all fractions), followed by pre-specified dose constraints for OAR doses exceeding tolerance (18%), or both indications (36%). For the OARs, PBT exceeded the tolerance dose in 112 fractions (57.4%), followed by the esophagus in 108 (55.4%), the spinal cord in 49 (25.1%), the heart in 29 (14.9%), the trachea in 28 (14.4%), the brachial plexus in 20 (10.3%), and normal lungs in 6 fractions (3.1%).

### 3.4. Locoregional Control

With a median follow-up of 6 months (range: 2–16), one patient had a locoregional in-field recurrence.

### 3.5. Acute Toxicity

Overall, the treatment was well tolerated. No grade 3 or higher toxicities (CTCAE v5.0) were observed. Four of thirteen patients (30.8%) developed grade 2 toxicities and three cases of grade 2 fatigue (in two patients who were receiving concurrent osimertinib and one who was receiving carboplatin plus paclitaxel). One patient developed grade 2 esophagitis (no concurrent systemic treatment), one developed grade 2 pneumonitis (concurrent osimertinib), and one experienced grade 2 cough (concurrent carboplatin plus paclitaxel).

## 4. Discussion

SBRT has established itself as a safe and effective treatment for peripheral lung tumors, showing minimal grade 4 and higher toxicities [11]. However, when extending the application to central tumors within the “no-fly zone” defined by the Radiation Therapy Oncology Group (RTOG), particularly with aggressively hypofractionated schedules, the landscape changes, leading to unacceptably high rates of high-grade toxicity [12]. Even less aggressively hypofractionated schedules have demonstrated modestly high rates of toxicities, especially concerning centrally located OARs. The HILUS trial further underscores the challenges, reporting a high risk of treatment-related death, particularly hemoptysis, reaching 15% for tumors situated 1 cm from the proximal bronchial tree (PBT) when treated with a hypofractionated schedule of 56 Gy in eight fractions (7 Gy per fraction). The PBT/trachea maximal dose (D_0.02cc_) was identified as a key predictor for respiratory tract hemorrhage [13]. Furthermore, Haseltine et al. reported a grade 5 toxicity rate of 33.3% (half of them due to acute hemorrhage) for patients treated with a total dose of 45 to 50 Gy in five fractions (9–10 Gy per fraction) [14].

**Figure 3:**
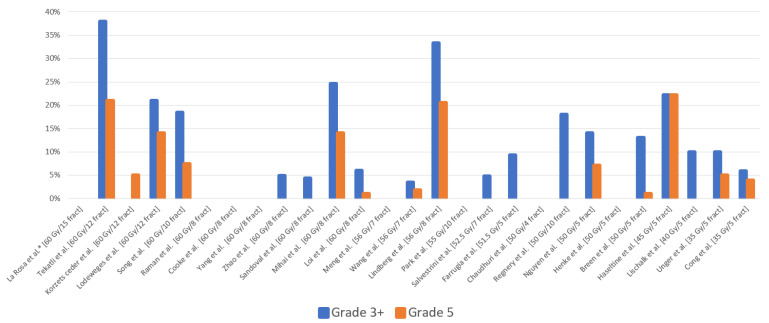
Treatment-related toxicities associated with hypofractionated schedules from this study and other case series, showing the median total dose and median number of fractions for each study [9,13,14,15,16,17,18,20,21,23,24,25,26,27,28,29,30,31,32,33,34,36,38,40,42,43]. * Current study. fract = fractions. Gy = Gray.

Different hypofractionated schedules have been utilized for ultracentral tumors (Supplementary Table S1) [9,13,14,15,16,17,18,19,20,21,22,23,24,25,26,27,28,29,30,31,32,33,34,35,36,37,38,39,40,41,42,43], but treatment-related toxicities remain concerning (Figure 3) [9,13,14,15,16,17,18,20,21,23,24,25,26,27,28,29,30,31,32,33,34,36,38,40,42,43]. Notably, two prior retrospective series detailed their experience with a more conservative hypofractionation schedule, administering a total of 60 Gy in 12 fractions (5 Gy per fraction), utilizing daily cone-beam computerized tomography (CBCT) for online setup and position verification. Lodeweges et al. reported a 72-patient series and described a grade 3+ toxicity of 21% and a fatal outcome secondary to bronchopulmonary hemorrhage of 14%. All these patients had PTVs overlapping with the PBT. Moreover, autopsies performed on two patients demonstrated bronchi-vascular fistulae. Interestingly, they found an association between the mean BED_3_ and the main bronchus of ≥91 Gy and an increasing risk of fistualization [33]. Tekatli et al. using this same fractionation schedule (60 Gy/12 fractions; 47 patients), reported a grade 3+ toxicity of 38%, with a grade 5 treatment-related toxicity of 21% (13% due to bronchopulmonary hemorrhage) [18]. In this series, the median maximum doses to the PBT and trachea on the original treatment plans were 63.4 Gy and 41.5 Gy (ranges: 62.5–66.3 Gy and 1–66.9 Gy), respectively. In our series, we adopted a more prolonged hypofractionation schedule, delivering 60 Gy in 15 fractions, which is a departure from the more aggressive regimens in prior studies. Crucially, our treatment methodology involved mid-inspiration breath hold, ensuring alignment between planning constraints and the delivered dose to central OARs. This approach stands in contrast to radiotherapy delivery methods that aim to reduce respiratory excursion, potentially leading to a dose cloud surpassing OAR constraints during treatment.

Moreover, in the context of discussing contributory factors to treatment complications, this cohort was a relatively old population (median age of 70 years), with a large treatment volume for radiation (up to 211.81 cc), with a significant portion being smokers or ex-smokers (77%). Our study population reflects a demographic profile commonly associated with increased vulnerability to side effects. Additionally, a subset of patients with a history of prior RT to the thoracic region (15.4%), pre-existing lung diseases (15.4%), and a substantial proportion on antiplatelets or anticoagulants (38.5%) introduce further complexity to our analysis. These risk factors, collectively considered, underscore the importance of a nuanced interpretation of treatment outcomes, as the interplay of these variables could potentially contribute to variations in observed toxicities.

The implementation of daily on-table MRgART imaging and recontouring uncovered noteworthy variations in PBT, trachea, and esophagus doses, surpassing the original planning directives in 57.4%, 14.4%, and 55.4%, respectively, of the total daily fractions. Without the ability to perform plan adaptation, the IGRT set-up alone results in a potentially significantly different planned vs. treated total dose distribution and potentially accounts for the increased high-grade treatment-related toxicities in other series.

Recently, the International Stereotactic Radiosurgery Society (ISRS) published a meta-analysis and a practical guideline on radiotherapy for ultracentral lung tumors, where they included studies with a variety of doses and fractionation schedules, ranging from 30 to 70 Gy in 3–10 fractions, and including 50 Gy/5 fractions, 60 Gy/8 fractions, and 60 Gy/12 fractions as the most common recommendations [44]. They recommend a total dose of 60 Gy in 8 or 15 fractions for ultracentral metastases and primary lung tumors. These hypofractionated schedules are associated with high local control rates (2-year 89–95%) and reduced treatment-related toxicities. In our institution, we have adopted the 60 Gy in 15 fractions schedule for ultracentral tumors, based on these recommendations and currently available data. Our early outcomes provide support for this practice paradigm [44,45,46].

Variability in daily anatomy (displacement or tumor size changes), especially in ultracentral thoracic tumors, must be taken into account as it is crucial to the delivery of accurate and precise RT. Most of the published series on ultracentral tumors have used cone-beam computerized tomography (CBCT), but this IGRT method could potentially be insufficient since intrafraction motion is not assessed and daily on-table adaptation is not utilized. This could account for the significant variation in treatment-related toxicities observed in retrospective and prospective studies in the published literature (Supplementary Table S1). In our study, the utilization of MRgART allowed for superior soft tissue visualization of the primary lung tumor as well as the mediastinal lymph nodes, gating of the treatment delivery to allow a reduced dose to the central OARs, as well as the ability to perform adaptive re-planning, which was needed and utilized for all 195 fractions. Changes in tumor anatomy and/or location during treatment required adaptation to avoid target undercoverage in 46% of cases as a sole indication, and in another 36%, both target coverage improvement and OAR dose reduction drove the need for adaptation.

MRgART outcomes for ultracentral tumors have been described in two retrospective series, including a mix of primary and metastatic tumors. (Supplementary Table S1). Moreover, a phase 1 clinical trial has shown the feasibility and safety of stereotactic online MRgART for oligometastatic or primary lung tumors with an ultracentral location, with no grade 3 or higher treatment-related toxicities reported [26]. As this platform allows for gated treatment, no ITV expansion was required. In these studies, a GTV expansion of 3 mm was used to create the PTV in one, and in the other, a 2 mm margin was used to create a clinical target volume (CTV) and then a 3 mm margin expansion from the CTV to create the PTV. In our series, we used a 5 mm expansion from the GTV to create the PTV. Sandoval et al. reported no acute toxicities in the 38 patients they treated (the most common fractionation schedules were 60 Gy in 8 and 60 Gy in 15 fractions), while in a study by Regnery et al., the most common fractionation schedule was 50–60 Gy in 10 fractions, and 91% [277/303] of the total fractions needed to be adapted. They reported 2 patients (of 16) with Grade 3+ toxicity, including 1 esophagitis G3 and 1 bronchial bleeding G4 [40,42]. In our case, no acute Grade 3+ toxicity was seen. A recently published phase 3 clinical trial has demonstrated the advantages in terms of event-free survival when combining SBRT with immune checkpoint inhibitors versus SBRT alone for patients with treatment-naïve early-stage NSCLC or those with recurrent lung parenchymal tumors (without lymph node involvement). It is important to note that there were no severe adverse effects (CTCAE v.5.0 grade 3+) associated with SBRT in this study. However, it is worth mentioning that the trial excluded patients with ultracentral tumors. Consequently, the relationship between this treatment approach and tumors located in ultracentral regions remains unestablished. Therefore, extra caution is advised when considering this treatment for patients with tumors in such locations [47].

This study has several inherent limitations, including its retrospective and single-institutional nature, limited follow-up, and heterogenous patient mix. Despite the limited number of patients included (13), the number of delivered fractions, which were individually analyzed (195), provides a robust understanding of the need for adaptation and the potential impact of this in minimizing or eliminating the most dreaded complication of broncho-vascular fistualization.

## 5. Conclusions

In our institutional study, the use of hypofractionated MRgART for ultracentral lung tumors has yielded highly promising outcomes. The application of a meticulously designed dose and fractionation schedule of 60 Gy in 15 fractions, coupled with daily online adaptation, demonstrated a remarkable absence of high-grade toxicities. This innovative approach not only ensured the safety of the treatment but also facilitated a more precise total dose calculation for OARs. The unique capability of MRgART to dynamically adapt to the anatomical shifts of internal structures and changes in target volumes throughout the treatment timeline played a crucial role. This adaptability not only improved target volume coverage but also effectively mitigated potential adverse effects on surrounding tissues, setting a new standard for the comprehensive and patient-centric management of ultracentral lung tumors.

## Figures and Tables

**Table 1:** Patients and disease characteristics of ultracentral thoracic tumors treated accelerated hypofractionated MRgART.

Age	Gender	Smoking Status	Anti-Platelets Anticoagulant	COPD, Asthma, or Other Lung Disease	Primary	Disease Status	Site	reRT	GTV (cc)	PTV (cc)	Concurrent Systemic Therapy	G2+ Toxicity
67	M	Ex-smoker	Yes	No	ADC	OP	11L	No	18.71	54.05	Pembrolizumab	-
84	M	Ex-smoker	Yes	No	ADC	OP	LLL	Yes	31.59	64.81	Durvalumab	-
85	M	Ex-smoker	Yes	Yes	SCC	OR	7	No	20.63	67.15	Carboplatin and Paclitaxel	Cough (G2) Fatigue (G2)
75	F	Never	No	No	SCC	OP	LUL + 10L	No	31.11	68.82	Osimertinib	Pneumonitis (G2) Fatigue (G2)
81	F	Ex-smoker	Yes	No	SqCC	OP	7 + RUL	No	26.5	68.56	Paclitaxel	-
78	M	Smoker	No	No	ADC	OP	11L	No	0.54	3.87	Capmatinib	-
50	M	Ex-smoker	No	No	ADC	OP	4R	No	4.58	15.83	None	-
56	M	No	No	No	Melanoma	OP	2R, 11 + RUL	No	20.81	58.25	Nivolumab	-
46	F	Smoker	No	No	ADC	OP	LUL	No	46.95	99.71	Osimertinib	Fatigue (G2)
71	M	Smoker	No	No	ADC	OP	RUL	No	121.65	211.81	None	-
59	M	Smoker	No	Yes	ADC	OP	RUL	No	100.76	173.36	Atezolizumab	-
68	F	Never	No	No	Carcinosarcoma	OR	4R + RUL	No	1.6	7.94	None	Esophagitis (G2)
70	M	Smoker	Yes	No	ADC	OP	4R	No	11.32	31.36	None	-

M = male; F = female; ADC = adenocarcionoma; SCC = small cell carcinoma; SqCC = squamous cell carcinoma; P = primary; OP = oligo-progressive; OR = oligo-recurrent; reRT = re-irradiation; cc = cubic centimeter; LUL = left upper lobe; RUL = right upper lobe; LLL = left lower lobe; GTV = gross tumor volume; PTV = planning target volume.

## Data Availability

The source data presented in this study are available on request from the corresponding author. The source data are not publicly available due to patient’s privacy.

## Supplementary Materials

The following supporting information can be downloaded at https://www.mdpi.com/article/10.3390/tomography10010013/s1. Table S1: Studies on ultracentral lung tumors treated with stereotactic body radiaiton therapy (SBRT) or hypofractionated schedules.
