# Supplementary material for: Accelerated Hypofractionated Magnetic Resonance Guided Adaptive Radiation Therapy for Ultracentral Lung Tumors
Source: Tomography. 2024 Jan 17;10(1):169–80. doi: 10.3390/tomography10010013 (PMC10820032; doi:10.3390/tomography10010013)
Supplement: Supplementary file 1 [file tomography-10-00013-s001.zip › tomography-2674764-supplementary.pdf]

Supplementary Table S1. Studies on ultracentral lung tumors treated with stereotactic body radiaiton therapy (SBRT) or hypofractionated schedules.

Reference	n	Patients Included	Dose	# Fx	Freq	Platform used	Breath-induced motion Assesment	Motion Assesment	Planning simulation imaging slice thickness	Target definition	Daily positioning and setup	RT technique	Outcomes	Toxicity
Song et al. 2009 [1] -R-	9	NSCLC (Early-stage)	40 - 60	3 - 4	D	NR	Active breathing control Abdominal compression RPM system (gating)	4DCT	NR	GTV + (5 mm laterally and 10 mm longitudinally) = PTV	CBCT Fluoroscopy	IMRT	OS2y 50% LC2y 84.9%	G3+ 33% Bronchial stricture (partial or complete) in 8 patients.
Unger et al. 2010 [2] -R-	20	Primary Metastatic	30-40	5	NR	CyberKnife	Synchrony Respiratory Motion Tracking System	BH	1.25-mm	GTV = PTV	NR	Robotic	OS1y 54% LC1y 63%	G2 esophagitis 1 patient G3 pneumonitis 1 patient G5 fistula (1 patient)
Chaudhuri et al. 2015 [3] -R-	7	Primary Metastatic	50	4 / 5	D	Trilogy TrueBeam CyberKnife	NR	4DCT	NR	ITV + 5 mm = PTV	kV X-ray CBCT	IMRT Robotic	OS2y 80% Primary NSCLC LC2-y 100%	No G2+ toxicity
Haseltine et al. 2016 [4] -R-	18	Primary Metastatic	45 / 50	5	EoD	NR	NR	4DCT	NR	GTV + motion = ITV ITV + 2-3 mm = CTV CTV + 5 mm = PTV	NR	IMRT	Overall (including non ultracentral) OSy 63.9% LC2y 77.4%	G5 toxicity 22.2% (4)
Lischalk et al. 2016 [5] -R-	20	Metastatic	35 - 40	5	D	CyberKnife	Synchrony Respiratory Motion Tracking System	NR	NR	GTV + 5 mm = PTV	NR	Robotic	LC1y 70% OS1y 75%	acute G2+ 1 patient (esophagitis) late G2+ 30% (G2 [atelectasias 3, bronchitis 1], G3 [pneumonitis 1], G4 [atelectasia 1])
Tekatli et al. 2016 [6] -R-	47	NSCLC (primary and recurrent)	60	12	4/week	NR	NR	4DCT	NR	NR	CBCT	VMAT	mOS 15.9m; eOS2y 20.1%	G3+ 38% (G3: pneumonitis 11%, dyspnea 6%, hemoptysis 4% and chest wall pain 4%; G4: hemoptysis 2%; G5: hemorrhage 13%, respiratory failure 2%, others 2%).
Chang et al. 2018 [7] -R-	46	Primary Metastatic	NR	5	EoD	Synergy	NR	4DCT	NR	GTV on 0% (peak inspiratory) GTV 50% (peak expiratory) GTV max intensity projection ITV= sum of GTV's	CBCT	IMRT VMAT	OS2y 50.4%	2y-G3+ 8.7%

										ITV + 5 mm = PTV				
Raman et al. 2018 [8] -R-	16	Primary Metastatic	48 - 60	3 - 10	D/EoD	NR	Abdominal compression	4DCT	NR	GTV = CTV ITV + 5 mm = PTV	CBCT	IMRT VMAT	mOS 23.8m (non metastatic) LC2y 100%	G2+ 7.9% No G4+
Korzets ceder et al. 2018 [9] -R-	20	Primary Metastatic	NR	NR	NR	TrueBeam	NR	4DCT	NR	ITV + 3-4 mm = PTV	CBCT	IMRT VMAT	Not specified for ultracentral	G5 1 patient No other toxicity reported
Lenglet et al. 2019 [10] -R-	77	Primary (early and advanced)	40 - 60	3 - 8	NR	CyberKnife TrueBeam Tomotherapy	Tracking Abdominal compresion (for lower lobes)	4DCT	3-mm	GTV/ITV + 5 mm = PTV	NR	Robotic VMAT	mOS 24m, mPFS 18m, LC18m	G3+ 7.8% (3 pneumonitis, 2 myocardial infarction amd 1 bronchial stricture)
Cong et al. 2019 [11] -R-	51	NSCLC (locally advanced)	35	5	D	CyberKnife	Tracking	4DCT	3-mm	ITV + 3 mm = PTV	NR	IMRT	local recurrence 47.1%	G5 3.9% (2 patients)
Nguyen et al. 2019 [12] -R-	14	Early-stage NSCLC (13) Oligometastatic (1)	40 / 50 / 56 / 60	5 / 5 / 8 / 8	NR	Synergy	Abdominal compression	4DCT	2-mm	GTV + motion = ITV ITV + 5 mm = PTV	CBCT Fluoroscopy	NR	LC2y 89% eOS2y 76%, eOS5y 33%	G2+2y 57.6%
Park et al. 2019 [13] -R-	8	Primary (NSCLC, 3) Consolidative (SCLC, 1) Recurrence (4)	50 - 60	10	NR	NR	Gating (RPM system) Compression belt	4DCT	2-mm 3-mm	GTV = CTV CTV + 5-7 mm = PTV	NR	NR	Respose rate 87.5% Disease control rate 100%	G2 esophagitis (1) No G3+ reported
Meng et al. 2019 [14] -R-	37	Early-stage NSCLC	48 - 60	5 - 10	D	CyberKnife	Synchrony tracking system Xsight spine-tracking system	4DCT	NR	ITV + 5 mm (for Xsight spine and variable dependin fiducial motion) = PTV	NR	Robotic	OS3y 68.7%; OS5y 57.4% PFS3y 47.7%; PFS5y 35.2%	No G3+ toxicity
Henke et al. 2019 [15] -P, phase 1-	5	Primary Metastatic	50	5	NR	MRIdian	Gating (cine MR)	BH	NR	GTV + 5 mm = PTV	NR	IMRT	OS 1y 60% LC1y 80%	Acute G3 (1 esophageal stricture) Acute G4 (1, heart failure)
Cooke et al. 2020 [16] -R-	22	Oligometastases	60	8	EoD	LINAC	NR	4DCT	2.5-mm	ITV + (3 to 5 mm) = PTV	CBCT	IMRT VMAT	LC1y 94.1% OS1y 84.4%	NO acute G3+ toxicity Acute G2 13.6% (1 dysphagia, 2 radiation pneumonitis)

														Late G2 fatige 4.5% (1 patient)
Wang et al. 2020 [17] -R-	88	Primary Metastatic	45 - 60	5 - 15	EoD	NR	NR	4DCT	NR	ITV + (2-3 mm) = CTV CTV + 5 mm = PTV	CBCT kV Xray	IMRT VMAT	LC1y 87.8%; LC2y 81% OS1y 78.6%; OS2y 64.5%; OS3y 53.1%	G3: 6% for pneumonitis, main bronchi occlusion 1% and tracheobronchial perforation in 1% G4: tracheal necrosis 1% G5: hemoptysis 7%, pneumonitis 1% and respiratory failure 3%
Yang et al. 2020 [18] -R-	21	Primary NSCLC Metastatic NSCLC	60	8	D	TrueBeam Edge	NR	4DCT	NR	ITV + 5 mm = PTV	CBCT	VMAT	mOS 15m mPFS 12m; PFS1y 71.1%; PFS2y 64% LC1y 92.9%; LC2y 92.9%	G2 (4 pneumonitis, 1 esophagitis, 1 myelosuppression) NO G3+
Zhao et al. 2020 [19] -R-	41	Primary Metastatic	60	8	D	NR	Free-breathing	4DCT	NR	ITV + 5 mm = PTV	CBCT kV Xray	3DCRT IMRT VMAT	LC1y 97.8%; LC2y 84.5% OS 29.3%	G3 (1 dyspnea and 1 hemoptysis) No G4+
Breen et al. 2021 [20] -R-	110	Primary NSCLC	48 / 60	4 / 8	D	LINAC	NR	4DCT	2-mm	GTV + motion = iGTV iGTV + 5mm = PTV	CBCT	3DCRT VMAT	OS1y 78%, OS2y 57%, OS5y 32%	G2+ 18% (acute); 27% (late) G5 4%
Farrugia et al. 2021 [21] -R-	43	NSCLC	50-60	5	2/week	NR	Gating, abdominal compression	NR	NR	NR	NR	3DCRT VMAT	Local failure 7%, regional failure 11.6%, distant failure 7% eOS2y 53.3%	Acute G3+ 30.2% G3 9.3% (1 cough, 2 chest wall pain and 1 dysphagia)
Lindberg et al. 2021 [22] -P-	65	Primary Metastatic	56	8	E/1-3d No >4/ week	LINAC	Abdominal compression	4DCT	≤3-mm	GTV = CTV CTV + 5-10 mm = PTV	CBCT	IMRT VMAT	LC2y 83%	G3+ 33.8% (22) G5 15.4% (10; 8 hemorrhage, 1 fistula, 1 penumonitis)
Lodeweges et al. 2021 [23] -R-	72	Primary Metastatic	60	12	4 /week	NR	NR	4DCT	NR	ITV + 3 mm = PTV	CBCT	IMRT VMAT	OS1y 77%; OS2y 52% LC1y 98%; LC2y 85%	G3+ 21% G5 14% (all hemorrhage)
Loi et al. 2021 [24] -R-	109	Oligometastases	45 – 70	5 – 10	D	TrueBeam	NR	4DCT	1.5-mm	ITV + 5 mm = PTV	CBCT	VMAT	LC1y 88%; LC2y 78% DMFS1y 54%; DMFS2y 35% PFS1y 53%; PFS2y 30%	G3+ 5% G5 1 esophagitis

													OS1y 88%;<br>OS2y 55%	
Sidiqi et al.<br>2021 [25]<br>-R (abstract)-	30	NR	40 – 50	5	NR	NR	NR	NR	NR	NR	NR	NR	LC1y 92.3%,<br>LC2y 82.1%<br>OS1y 89.1%,<br>OS2y 65.7	G3+ 13.3% (2 pneumotinits, 1 pleural effusion, 1 large cavitary lesion)<br>G5 3.3% (1, hemoptysis)
Mihai et al.<br>2021 [26]<br>-R-	57	Primary<br>Oligometastatic	40 – 60	4 –<br>10	NR	Trilogy<br>TrueBeam	Gating: RPM system<br>+/- DIBH	4DCT	NR	GTV + motion = ITV<br>ITV/GTV + 5 mm = PTV	CBCT	IMRT	LC2y 92%,<br>LC 3y 88.5%, LC4y 79.8%<br>OS2y 55.1%, OS3y 49.3%, OS4y 41.2%	Acute: G3 3.5% (1 fatigue; 2 pneumonitis)<br>Late: G3 5.4% (1 fatigue, 1 dyspnea, 1 aspiration)<br>G5 14% (5 hemoptisis, 2 pneumonia, 1 COPD exacerbation)
Guillaume et al.<br>2022 [27]<br>-R-	74	Primary<br>Metastatic	NR	5 –<br>10	E/2d	CyberKnife<br>Synergy<br>Versa HD	Tracking	4DCT	2-mm	GTV/ITV + 5 mm = PTV	NR	NR	LC1y 96.7%<br>LC2y 87.6%<br>mOS 31m	G3 2.7% (pneumonitis and esophagitis)<br>No G4+
Salvestrini et al.<br>2022 [28]<br>-R-	126	Primary<br>Metastatic	45 – 60	5 – 7	NR	CyberKnife	NR	NR	NR	GTV + 5 mm = PTV	NR	Robotic	OS1y 75%;<br>OS2y 58%;<br>OS5y 23%<br>PFS1y 63%;<br>PFS2y 41%,<br>PFS5y 15%	Acute G3 1 (dyspnea)<br>Late G3 4% (1 pain, 4 dyspnea)
Wang et al.<br>2022 [29]<br>-R-	58	Primary<br>Metastatic	56	6 – 8	NR	CyberKnife	Fiducial-tracking system	4DCT	NR	GTV + 5 mm = PTV	NR	Robotic	OS1y 94.7%;<br>OS2y 75.0%;<br>and OS5y 45.0%	G3+ 53.5%<br>G5 1 patient (hemoptysis)
Sandoval et al.<br>2023 [30]<br>-R-	38	Primary<br>Metastatic	50 – 60	3 –<br>15	NR	MRIdian	Gating<br>(DIBH)<br>tracking	BH	NR	GTV + 3 mm = PTV	MR	IMRT	LC 94.7%	no G3+ acute toxicity<br>Overall (including C and UC):<br>G3 late toxicity 2 (1 pneumonitis, 1 esophagitis)
Song et al.<br>2023 [31]<br>-R-	27	NSCLC	56 / 60	7 / 10	D	NR	NR	4DCT	NR	GTV + motion = ITV<br>ITV + 5 mm = PTV	CBCT	IMRT	mOS 52m<br>mPFS 38m	G3+18.5%5<br>G5 7.4%<br>(1 bronchial obstruction, 1 esophageal perforation)
Regnery et al.<br>2023 [32]<br>-R-	16	Early NSCLC (4)<br>Oligometastatic (12)	50 - 60	10	D	MRIdian	Gating	BH	NR	GTV + 2 mm = CTV<br>CTV + 3 mm = PTV	MR	IMRT	OS2y 67%<br>PFS2y 37%	G3+ 12.5%<br>(1 esophagitis G3,<br>1 bronchial bleeding G4)
La Rosa et al.<br>*2023**<br>*-R-*	13	Primary<br>Metastatic	60	15	D	MRIdian	Gating (cine-MR)	4DCT	3-mm	GTV + 5 mm = PTV	MR	IMRT	-	No G3+ acute toxicity

*N=number of patients; "# Fx"= number of fractions; Freq=frequency; RT=radiation therapy; -R-=retrospective; -P-=prospective; D=daily; NR=not reported; EoD=every other day; NSCLC=non-small-cell lung cancer; GTV=gross tumor volume; ITV=internal target volume; CTV=clinical target volume; PTV=[lanning target volume; mm=millimeter; G=grade; 4DCT=four-dimensional computed tomography; CBCT=cone-beam computed tomograph;y kV=kilovoltage; OS=overall survival; LC=local control; PFS=progression-free survival; m=media;n "m"=months; "y"=years; 3DCRT=three-dimensional conformal radiation therapy; IMRT=intensity-modulated radiation therapy; VMAT=volumetric modulated arc therapy; *current study.*

References

1. Song, S.; Choi, W.; Shin, S.; Lee, S.; Ahn, S.; Kim, J.; al., e. Fractionated stereotactic body radiation therapy for medically inoperable stage I lung cancer adjacent to central large bronchus. *Lung cancer (Amsterdam, Netherlands)* **2009**, *66*, 89-93, doi:10.1016/j.lungcan.2008.12.016.
2. Unger, K.; Ju, A.; Oermann, E.; Suy, S.; Yu, X.; Vahdat, S.; Subramaniam, D.; William Harter, K.; Collins, S.P.; Dritschilo, A.; et al. CyberKnife for hilar lung tumors: report of clinical response and toxicity. *Journal of Hematology & Oncology* **2010**, *3*, 39, doi:10.1186/1756-8722-3-39.
3. Chaudhuri, A.A.; Tang, C.; Binkley, M.S.; Jin, M.; Wynne, J.F.; von Eyben, R.; Hara, W.Y.; Trakul, N.; Loo, B.W., Jr.; Diehn, M. Stereotactic ablative radiotherapy (SABR) for treatment of central and ultra-central lung tumors. *Lung Cancer* **2015**, *89*, 50-56, doi:10.1016/j.lungcan.2015.04.014.
4. Haseltine, J.M.; Rimner, A.; Gelblum, D.Y.; Modh, A.; Rosenzweig, K.E.; Jackson, A.; Yorke, E.D.; Wu, A.J. Fatal complications after stereotactic body radiation therapy for central lung tumors abutting the proximal bronchial tree. *Pract Radiat Oncol* **2016**, *6*, e27-33, doi:10.1016/j.prro.2015.09.012.
5. Lischalk, J.W.; Malik, R.M.; Collins, S.P.; Collins, B.T.; Matus, I.A.; Anderson, E.D. Stereotactic body radiotherapy (SBRT) for high-risk central pulmonary metastases. *Radiat Oncol* **2016**, *11*, 28, doi:10.1186/s13014-016-0608-8.
6. Tekatli, H.; Haasbeek, N.; Dahele, M.; De Haan, P.; Verbakel, W.; Bongers, E.; al., e. Outcomes of Hypofractionated High-Dose Radiotherapy in Poor-Risk Patients with "Ultracentral" Non-Small Cell Lung Cancer. *Journal of thoracic oncology : official publication of the International Association for the Study of Lung Cancer* **2016**, *11*, 1081-1089, doi:10.1016/j.jtho.2016.03.008.
7. Chang JH; Poon I; Erler D; Zhang L; P., C. The safety and effectiveness of stereotactic body radiotherapy for central versus ultracentral lung tumors. *Radiother Oncol.* **2018**, *129*, 277-283.
8. Raman, S.; Yau, V.; Pineda, S.; Le, L.W.; Lau, A.; Bezjak, A.; Cho, B.C.J.; Sun, A.; Hope, A.J.; Giuliani, M. Ultracentral Tumors Treated With Stereotactic Body Radiotherapy: Single-Institution Experience. *Clin Lung Cancer* **2018**, *19*, e803-e810, doi:10.1016/j.cllc.2018.06.001.
9. Korzets ceder, Y.; Fenig, E.; Popvtzer, A.; Peled, N.; Kramer, M.R.; Saute, M.; Bragilovsky, D.; Schochat, T.; Allen, A.M. Stereotactic body radiotherapy for central lung tumors, yes we can! *Radiation Oncology* **2018**, *13*, 77, doi:10.1186/s13014-018-1017-y.
10. Lenglet, A.; Campeau, M.P.; Mathieu, D.; Bahig, H.; Lambert, L.; Vu, T.; Roberge, D.; Bilodeau, L.; Filion, E. Risk-adapted stereotactic ablative radiotherapy for central and ultra-central lung tumours. *Radiother Oncol* **2019**, *134*, 178-184, doi:10.1016/j.radonc.2019.01.035.
11. Cong, Y.; Sun, B.; Wang, J.; Meng, X.; Xuan, L.; Zhang, J.; Liu, J.; Shen, G.; Wu, S. Outcomes and toxicity of stereotactic body radiation therapy for advanced stage ultra-central non-small cell lung cancer. *Thorac Cancer* **2019**, *10*, 1567-1575, doi:10.1111/1759-7714.13105.
12. Nguyen, K.N.B.; Hause, D.J.; Novak, J.; Monjazeb, A.M.; Daly, M.E. Tumor Control and Toxicity after SBRT for Ultracentral, Central, and Paramediastinal Lung Tumors. *Practical Radiation Oncology* **2019**, *9*, e196-e202, doi:https://doi.org/10.1016/j.prro.2018.11.005.
13. Park, S.; Kim, Y.; Yoon, W.S.; Rim, C.H. A preliminary experience of moderate-intensity stereotactic body radiation therapy for ultra-central lung tumor. *International Journal of Radiation Biology* **2019**, *95*, 1287-1294, doi:10.1080/09553002.2019.1626026.
14. Meng, M.-B.; Wang, H.-H.; Zaorsky, N.G.; Sun, B.-S.; Zhu, L.; Song, Y.-C.; Li, F.-T.; Dong, Y.; Wang, J.-S.; Chen, H.-M.; et al. Risk-adapted stereotactic body radiation therapy for central and ultra-central early-stage inoperable non-small cell lung cancer. *Cancer Science* **2019**, *110*, 3553-3564, doi:https://doi.org/10.1111/cas.14185.
15. Henke, L.E.; Olsen, J.R.; Contreras, J.A.; Curcuru, A.; DeWees, T.A.; Green, O.L.; Michalski, J.; Mutic, S.; Roach, M.C.; Bradley, J.D.; et al. Stereotactic MR-Guided Online Adaptive Radiation Therapy (SMART) for Ultracentral Thorax Malignancies: Results of a Phase 1 Trial. *Adv Radiat Oncol* **2019**, *4*, 201-209, doi:10.1016/j.adro.2018.10.003.
16. Cooke, R.; Camilleri, P.; Chu, K.-Y.; O'Cathail, S.M.; Robinson, M.; Van Den Heuvel, F.; Hawkins, M.A. Stereotactic body radiotherapy for moderately central and ultra-central oligometastatic disease: Initial outcomes. *Technical Innovations & Patient Support in Radiation Oncology* **2020**, *13*, 24-30, doi:https://doi.org/10.1016/j.tipsro.2020.01.002.
17. Wang, C.; Rimner, A.; Gelblum, D.Y.; Dick-Godfrey, R.; McKnight, D.; Torres, D.; Flynn, J.; Zhang, Z.; Sidiqi, B.; Jackson, A.; et al. Analysis of pneumonitis and esophageal injury after stereotactic body radiation therapy for ultra-central lung tumors. *Lung Cancer* **2020**, *147*, 45-48, doi:10.1016/j.lungcan.2020.07.009.

18. Yang, D.; Cui, J.; Zhao, J.; You, J.; Yu, R.; Yu, H.; Jiang, L.; Li, D.; Xu, B.; Shi, A. Stereotactic ablative radiotherapy of 60 Gy in eight fractions is safe for ultracentral non-small cell lung cancer. *Thorac Cancer* **2020**, *11*, 754-761, doi:10.1111/1759-7714.13335.
19. Zhao, Y.; Khawandanh, E.; Thomas, S.; Zhang, S.; Dunne, E.M.; Liu, M.; Schellenberg, D. Outcomes of stereotactic body radiotherapy 60 Gy in 8 fractions when prioritizing organs at risk for central and ultracentral lung tumors. *Radiation Oncology* **2020**, *15*, 61, doi:10.1186/s13014-020-01491-w.
20. Breen, W.G.; Jeans, E.B.; Gergelis, K.R.; Garces, Y.I.; Park, S.S.; Merrell, K.W.; Peikert, T.D.; Mansfield, A.S.; Wigle, D.A.; Harmsen, W.S.; et al. Ablative radiotherapy for ultracentral lung cancers: Dosimetric, geometric, and volumetric predictors of outcomes and toxicity. *Radiotherapy and Oncology* **2021**, *158*, 246-252, doi:10.1016/j.radonc.2021.03.001.
21. Farrugia, M.; Ma, S.J.; Hennon, M.; Nwogu, C.; Dexter, E.; Picone, A.; Demmy, T.; Yendamuri, S.; Yu, H.; Fung-Kee-Fung, S.; et al. Exceeding Radiation Dose to Volume Parameters for the Proximal Airways with Stereotactic Body Radiation Therapy Is More Likely for Ultracentral Lung Tumors and Associated with Worse Outcome. *Cancers (Basel)* **2021**, *13*, doi:10.3390/cancers13143463.
22. Lindberg, K.; Grozman, V.; Karlsson, K.; Lindberg, S.; Lax, I.; Wersäll, P.; al., e. The HILUS-Trial-a Prospective Nordic Multicenter Phase 2 Study of Ultracentral Lung Tumors Treated With Stereotactic Body Radiotherapy. *Journal of thoracic oncology : official publication of the International Association for the Study of Lung Cancer* **2021**, *16*, 1200-1210, doi:10.1016/j.jtho.2021.03.019.
23. Lodeweges, J.; van Rossum, P.; Bartels, M.; van Lindert, A.; Pomp, J.; Peters, M.; al., e. Ultra-central lung tumors: safety and efficacy of protracted stereotactic body radiotherapy. *Acta oncologica (Stockholm, Sweden)* **2021**, *60*, 1061-1068, doi:10.1080/0284186X.2021.1942545.
24. Loi, M.; Franceschini, D.; Dominici, L.; Chiola, I.; Franzese, C.; D'Agostino, G.R.; Navarria, P.; Marzo, M.; Paganini, L.; Comito, T.; et al. Dose coverage impacts local control in ultra-central lung oligometastases treated with stereotactic radiotherapy. *Strahlenther Onkol* **2021**, *197*, 396-404, doi:10.1007/s00066-020-01687-9.
25. Sidiqi, B.U.; Eckstein, J.; Nosrati, J.D.; Baker, J.; Antone, J.; Malesevic, V.; Seetharamu, N.; Sharma, R.; Ghaly, M. Analysis of Toxicity and Local Control for Ultra-Central Lung Tumors Undergoing Stereotactic Body Radiation Therapy. *International journal of radiation oncology, biology, physics* **2021**, *111*, e424, doi:10.1016/j.ijrobp.2021.07.1211.
26. Mihai, A.M.; Armstrong, P.J.; Hickey, D.; Milano, M.T.; Dunne, M.; Healy, K.; Thirion, P.; Heron, D.E.; ElBeltagi, N.; Armstrong, J.G. Late Toxicity and Long-Term Local Control in Patients With Ultra-Central Lung Tumours Treated by Intensity-Modulated Radiotherapy-Based Stereotactic Ablative Body Radiotherapy With Homogenous Dose Prescription. *Clin Oncol (R Coll Radiol)* **2021**, *33*, 627-637, doi:10.1016/j.clon.2021.05.005.
27. Guillaume, E.; Tanguy, R.; Ayadi, M.; Claude, L.; Sotton, S.; Moncharmont, C.; Magné, N.; Martel-Lafay, I. Toxicity and efficacy of stereotactic body radiotherapy for ultra-central lung tumours: a single institution real life experience. *The British Journal of Radiology* **2022**, *95*, 20210533, doi:10.1259/bjr.20210533.
28. Salvestrini, V.; Duijm, M.; Loi, M.; Nuyttens, J.J. Survival and Prognostic Factors of Ultra-Central Tumors Treated with Stereotactic Body Radiotherapy. *Cancers (Basel)* **2022**, *14*, doi:10.3390/cancers14235908.
29. Wang, B.; Dong, Y.; Yu, X.; Li, F.; Wang, J.; Chen, H.; Niu, Z.; Song, Y.; Yuan, Z.; Tao, Z. Safety and Efficacy of Stereotactic Ablative Radiotherapy for Ultra-Central Lung Cancer. *Front Oncol* **2022**, *12*, 868844, doi:10.3389/fonc.2022.868844.
30. Sandoval, M.L.; Sim, A.J.; Bryant, J.M.; Bhandari, M.; Wuthrick, E.J.; Perez, B.A.; Dilling, T.J.; Redler, G.; Andreozzi, J.; Nardella, L.; et al. Magnetic Resonance-Guided Stereotactic Body Radiation Therapy/Hypofractionated Radiation therapy for Metastatic and Primary Central and Ultracentral Lung Lesions. *JTO Clin Res Rep* **2023**, *4*, 100488, doi:10.1016/j.jtocrr.2023.100488.
31. Song, X.; Zhao, L.; Jiang, N.; Ding, N.; Zong, D.; Zhang, N.; Wang, D.; Wen, J.; He, X.; Kong, C.; et al. Long-term outcomes in patients with central and ultracentral non-small cell lung cancer treated with stereotactic body radiotherapy: single-institution experience. *Curr Probl Cancer* **2023**, *47*, 100956, doi:10.1016/j.currproblcancer.2023.100956.
32. Regnery, S.; Katsigiannopulos, E.; Hoegen, P.; Weykamp, F.; Sandrini, E.; Held, T.; Deng, M.; Eichkorn, T.; Buchele, C.; Rippke, C.; et al. To fly or not to fly: Stereotactic MR-guided adaptive radiotherapy effectively treats ultracentral lung tumors with favorable long-term outcomes. *Lung Cancer* **2023**, *179*, 107175, doi:10.1016/j.lungcan.2023.03.011.
